# Triptolide decreases podocytes permeability by regulating TET2-mediated hydroxymethylation of ZO-1

**DOI:** 10.3389/ebm.2024.10051

**Published:** 2024-05-31

**Authors:** Yue-Wen Tang, Meng-Ya Jiang, Jia-Wei Cao, Feng Wan

**Affiliations:** ^1^ Department of Nephrology, Hangzhou TCM Hospital Affiliated to Zhejiang Chinese Medical University, Hangzhou, China; ^2^ Key Laboratory of Kidney Disease Prevention and Control Technology, Hangzhou, China

**Keywords:** Triptolide, TET2, hydroxymethylation, ZO-1, podocytes permeability

## Abstract

Podocyte injury or dysfunction can lead to proteinuria and glomerulosclerosis. Zonula occludens 1 (ZO-1) is a tight junction protein which connects slit diaphragm (SD) proteins to the actin cytoskeleton. Previous studies have shown that the expression of ZO-1 is decreased in chronic kidney disease (CKD). Thus, elucidation of the regulation mechanism of ZO-1 has considerable clinical importance. Triptolide (TP) has been reported to exert a strong antiproteinuric effect by inhibiting podocyte epithelial mesenchymal transition (EMT) and inflammatory response. However, the underlying mechanisms are still unclear. We found that TP upregulates ZO-1 expression and increases the fluorescence intensity of ZO-1 in a puromycin aminonucleoside (PAN)-induced podocyte injury model. Permeablity assay showed TP decreases podocyte permeability in PAN-treated podocyte. TP also upregulates the DNA demethylase TET2. Our results showed that treatment with the DNA methyltransferase inhibitors 5-azacytidine (5-AzaC) and RG108 significantly increased ZO-1 expression in PAN-treated podocytes. Methylated DNA immunoprecipitation (MeDIP) and hydroxymethylated DNA immunoprecipitation (hMeDIP) results showed that TP regulates the methylation status of the ZO-1 promoter. Knockdown of TET2 decreased ZO-1 expression and increased methylation of its promoter, resulting in the increase of podocyte permeability. Altogether, these results indicate that TP upregulates the expression of ZO-1 and decreases podocyte permeability through TET2-mediated 5 mC demethylation. These findings suggest that TP may alleviate podocyte permeability through TET2-mediated hydroxymethylation of ZO-1.

## Highlights

- Triptolide upregulates ZO-1 to decrease podocyte permeability in PAN-induced podocyte injury model.
- Triptolide regulates the methylation status of ZO-1 promoter in podocyte.
- Knockdown of TET2 decreases ZO-1 expression, accompanied by an increase in ZO-1 promoter methylation levels.

## Impact statement

Proteinuria is one of the most common manifestations of chronic kidney disease (CKD) and also one of the most important independent risk factors for CKD progression. Podocyte injury is the major cause of proteinuria. Zonula occludens 1 (ZO-1) maintains podocyte structure by connecting the slit diaphragm (SD) proteins to the actin cytoskeleton. Numerous studies have confirmed that the expression of ZO-1 is decreased in CKD. Therefore, clarifying the regulatory mechanism of ZO-1 is of great significance. Triptolide (TP) can alleviate proteinuria through various mechanisms. In this study, we aimed to clarify the mechanism by which TP regulates ZO-1 from the novel perspective of epigenetics. Accordingly, a puromycin aminonucleoside (PAN)-induced podocyte injury model was developed and treated with TP. The results showed that TP upregulates the expression of ZO-1 and decreases podocyte permeability through TET2-mediated hydroxymethylation of the ZO-1 promoter. Our study reveals the novel mechanism by which TP can reverse podocyte damage.

## Introduction

Chronic kidney disease (CKD) is a highly progressive disease, which has becoming one of the leading causes of death in the 21st century [1]. Proteinuria is not only an early clinical manifestation of CKD, but also an independent prognostic risk factor for CKD progression [2]. Podocytes, also known as glomerular epithelial cells, are highly differentiated cells that are attached to the glomerular basement membrane (GBM) and located in the outermost layer of the GBM [3]. Podocytes are connected to each other by the slit diaphragm (SD), and the integrity of this connection plays an important role in preventing renal protein loss. Podocyte injury or dysfunction can lead to proteinuria and glomerulosclerosis [4]. Therefore, effective prevention and treatment of podocyte injury is an important issue.

Zonula Occludens 1 (ZO-1) belongs to the uridine kinase family. It was the first tight junction proteins to be identified, and contains the PDZ, SH3 and uridine acid regions [5]. ZO-1 is mainly expressed on the cytoplasmic side of the foot process of glomerular podocytes near the SD and links the SD proteins through its PDZ domain to the actin cytoskeleton [6]. Accumulating studies have shown that the expression of ZO-1 is significantly reduced in CKD [7–9]. Therefore, the correct localization and expression of ZO-1 in the tight junction between the podocytes are particularly important for the maintenance of podocyte permeability.

Triptolide (TP) is a major active component of the medicinal plant *Tripterygium wilfordii* Hook. f., and is widely used for the treatment of inflammation and autoimmune disorders. Animal experiments have suggested that TP can relieve proteinuria by various mechanisms, including anti-inflammatory, and antioxidant effects, inhibition of epithelial mesenchymal transition, and promotion of autophagy [10–14]. However, whether TP can affect the expression of ZO-1 in podocytes remains unclear.

In the present study, we used a puromycin aminonucleoside (PAN)-induced podocyte injury model to explore the effect of TP on ZO-1 expression, podocyte permeability as well as to detect the methylation status of the promoter of ZO-1.

## Materials and methods

### Cell culture and treatment

Human conditionally immortalized glomerular podocytes (HPCs) were cultured in complete RPMI 1640 medium containing 10% fetal bovine serum (Gibco, Gaithersburg, MD, United States) and insulin-transferrin-selenium (1×, Thermo Fisher Scientific, Waltham, MA, United States) at 33°C in 5% CO_2_. After differentiation at 37°C for 10–14 days, the mature podocytes were randomly divided into three groups: Control, PAN-stimulated group (PAN, 50 μg/mL, GLPBIO), and TP intervention group (5 nM, Yuanye Bio-Technology Co., Ltd, Shanghai). Additionally, 5′-azacytidine (5-AzaC, 10 μM in phosphate-buffered saline [PBS], MCE, United States) or RG108 (Beyotime, Shanghai, China) were used as the DNA methyltransferase inhibitors. Lentivirus (Lv) carrying the TET2 short-hairpin RNA (shRNA) and control lentivirus (scramble Lv) were purchased from GeneChem (Shanghai, China). After incubation for 24–72 h, cells were collected for subsequent experiments.

### Detection of reactive oxygen species (ROS) level

HPCs were treated with PAN and TP for 24 h, then incubated with 10 μM DCFH-DA (Beyotime, Shanghai, China) for 30 min, followed by washing three times with PBS. Thereafter, the fluorescence intensity of DCFH-DA was detected using fluorescent microscopy.

### Permeability assay

The permeability assay was conducted as previously described [15]. Briefly, HPCs (1 × 10^5^/well/200 μL) were seeded in the upper layer of the transwell chamber (24 well plate, NEST Biotechnology Co. Ltd, Wuxi, China). Cells were treated with PAN (50 μg/mL) or TP (5 nM) after undergoing starvation. After 36 h, fluorescein isothiocyanate (FITC)-labeled bovine serum albumin (0.25 mg/mL, Solarbio, Beijing, China) was added to the upper chamber of the transwell chamber for 10 min at room temperature. The fluorescence intensity of the medium in the lower chamber, reflecting the podocyte monolayer permeability, was detected using a multifunctional enzyme-linked immunosorbent assay reader (excitation: 493 nm, emission: 550 nm; Thermo Fisher Scientific, Waltham, MA, United States).

### Quantitative real-time polymerase chain reaction (qRT-PCR)

Total RNA was extracted from HPCs using the TRIzol reagent and reverse-transcribed to cDNA using a Primescript™ reagent kit (TaKaRa, Kusatsu, Shiga, Japan). The primers used were as follows: ZO-1-Forward: 5′-CAA​CAT​ACA​GTG​ACG​CTT​CAC​A′, ZO-1-Reverse: 5′-CAC​TAT​TGA​CGT​TTC​CCC​ACT​C-3′; TET2-Forward: 5′-TCC​TGG​TGG​CAG​CTC​TGA​ACG-3′, TET2-Reverse: 5′-TGG​TGG​TGG​TGG​TGT​GGT​AGT​G-3’; GAPDH-Forward: 5′-ACC​ACA​GTC​CAT​GCC​ATC​AC-3′, GAPDH-Reverse: 5′-TCC​ACC​ACC​CTG​TTG​CTG​TA-3′. PCR was performed using a QuantStudio 5 real-time PCR system (Applied Biosystems, Waltham, MA, United States). GAPDH was used as an internal reference. Each experiment was performed in triplicate.

### Western blotting

HPCs were lysed using RIPA buffer containing a proteinase inhibitor cocktail at 4°C for 30 min and centrifuged at 10,000 × *g* for 10 min. The BCA reagent kit (Beyotime, Shanghai, China) was used to calculate the protein concentration in the supernatant, followed by mixing with SDS-PAGE sample loading buffer (2×) and boiling at 95°C for 10 min. Equal amounts of protein were separated using SDS-PAGE and transferred to nitrocellulose membranes. Membranes were blocked with 5% skim milk, and then incubated with primary antibodies, including TET2 (Abcam, Cambridge, UK, ab94580), ZO-1 (ABclonal, Wuhan, China, A0659), NOX4 (ProteinTech, Rosemont, IL, United States, 67,681-1-1g), and GAPDH (ProteinTech, Rosemont, IL, United States, 60,004-1-Ig) at 4°C overnight, followed by incubation with infrared-labeled anti-rabbit/mouse IgG antibody. Reactive bands were observed using an Odyssey infrared imaging system (LI-COR Biosciences, Lincoln, NE, United States).

### Immunofluorescence

Podocytes were fixed with 10% formalin for 10 min at room temperature, blocked with 5% goat serum, and incubated with rabbit anti-ZO-1 (ABclonal, Wuhan, China) at 4°C for 1 h. Podocytes were then incubated with FITC-conjugated goat anti-rabbit IgG (Thermo Fisher Scientific, Waltham, MA, United States) at 4°C for 1 h after washing with PBS three times. Cells were photographed under a fluorescence microscope (Olympus) after staining with DAPI.

### DNA isolation and shearing

DNA was isolated using the genome extraction kit (Genefist, Shanghai, China), according to the manufacturer’s protocol. The genome was broken into 200–1000 bp segments by ultrasonic fragmentation. The ultrasonic conditions were set as: sonicate 3-4 pulses of 10 s each at 50 W, followed by 30 s rest on ice between each pulse. The sonicated cell lysate was analyzed by agarose gel analysis before subsequent use.

### Methylated/hydroxymethylated DNA immunoprecipitation (MeDIP/hMeDIP)

Methylated DNA was isolated from sonicated DNA (1.0 μg) using the EpiQuik™ Methylated DNA Immunoprecipitation Kit (Epigentek, Farmingdale, NY, United States), according to the manufacturer’s protocol. The EpiQuik™ Hydroxymethylated DNA Immunoprecipitation Kit (Epigentek, Farmingdale, NY, United States) was used to extract hydroxymethylated DNA from the sonicated DNA (0.5 μg) in each group. Meanwhile, total sonicated DNA was used as the input DNA for equal loading.

### Amplification of methylated and hydroxymethylated ZO-1

For DNA amplification, the PCR reactions were performed with a volume of 20 μL containing: TaKaRa Ex Taq^®^ (2×) 10 μL, forward primer (10 μM) 1 μL, reverse primer (10 μM) 1 μL, eluted DNA 2 μL, and ddH_2_O 6 μL. The primer sequences were as follows: Pro-ZO-1-Forward: 5′-AAC​GAG​AGC​AAC​GCT​TCT​GA-3′ and Pro-ZO-1-Reverse: 5′-GTC​CAC​TTG​CTC​CTC​GAC​AA-3′. All reactions were performed on a Veriti^®^ 96-Well Thermal Cycle (Thermo Fisher Scientific), and the PCR products were observed using a Gel Imaging System.

### Statistical analysis

Statistical analyses were performed using SPSS (version 20.0, IBM Corp., Armonk, NY, United States). Values are presented as mean ± standard deviation. Quantitative data were compared between the two groups using the student’s t-test. One-way analysis of variance was used for multiple group comparisons. *p*-value <0.05 was considered statistically significant.

## Results

### TP upregulates ZO-1 to decrease podocyte permeability

**FIGURE 1:**
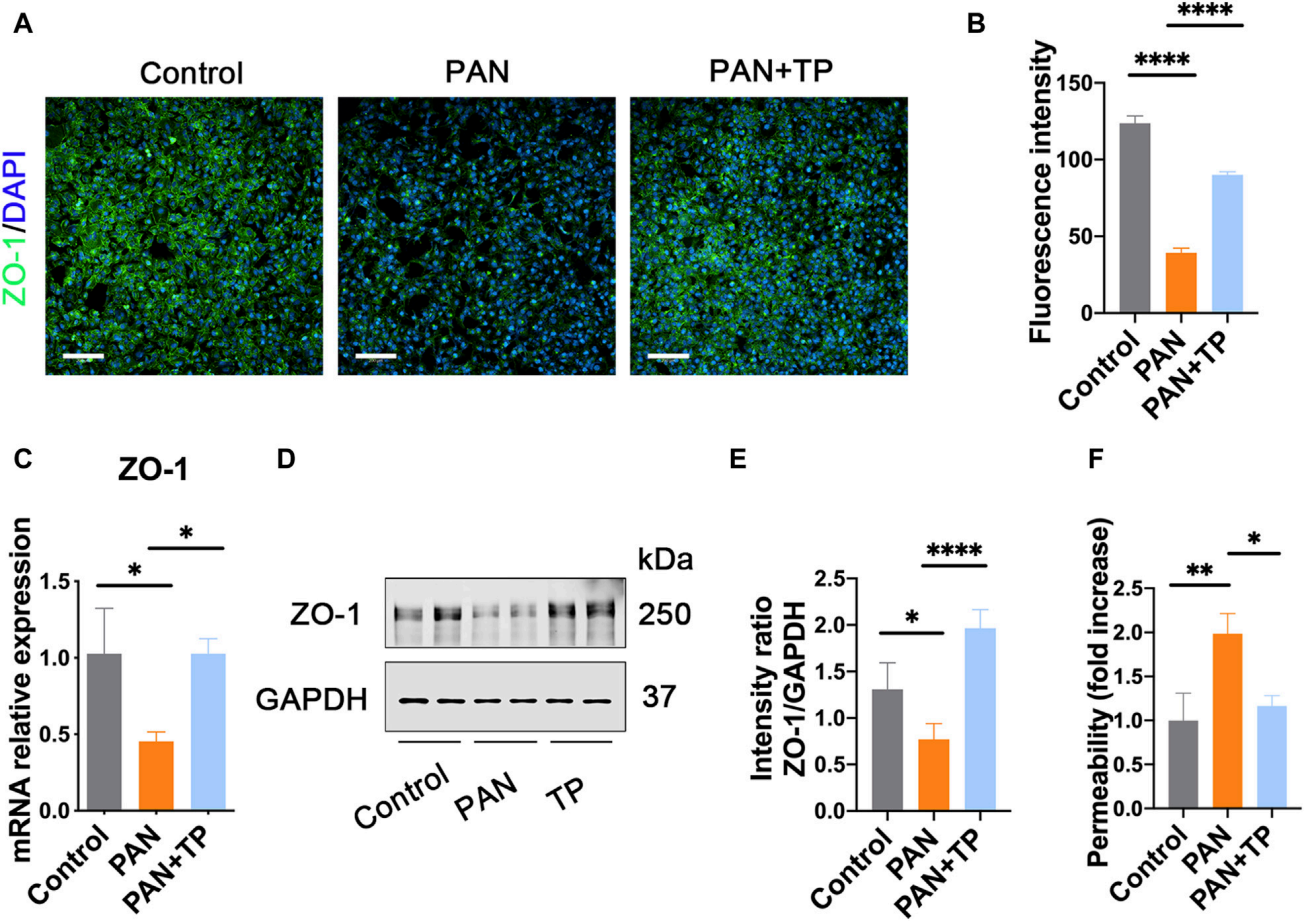
TP upregulates ZO-1 expression and decreases podocyte permeability in PAN-induced podocytes. **(A)** Immunofluorescence staining for ZO-1 in each group. Scale bar, 200 μm. **(B)** Semi-quantitative analysis of the fluorescence intensity of ZO-1. ****p < 0.0001. **(C)** The mRNA expression of ZO-1 in each group. Data are shown as mean ± SD. *p < 0.05. **(D)** The protein expression of ZO-1 in each group. **(E)** Relative protein level was calculated by the band intensity against GAPDH, respectively. *p < 0.05, ****p < 0.0001. **(F)** The podocyte permeability was detected using FITC-labeled bovine serum albumin in a transwell chamber. *p < 0.05, **p < 0.01.

PAN induces podocyte injury by causing decreased expression and abnormal distribution of SD proteins, including ZO-1 [16]. Similarly, in our study, we observed a decrease in the fluorescence intensity of ZO-1, along with its discontinuous distribution in PAN-stimulated podocytes (Figures 1A, B). ZO-1 mRNA and protein expression levels were also decreased (Figures 1C–E). However, TP treatment markedly increased the fluorescence intensity and upregulated ZO-1 expression levels. Next, we assessed podocyte permeability using a transwell chamber, and found that the permeability of PAN-treated podocytes was nearly twice of that of untreated podocytes (Figure 1F). However, TP intervention significantly decreased the podocyte permeability of the PAN-treated group. These findings suggest that TP can decrease podocyte permeability by upregulating ZO-1 expression.

**FIGURE 2:**
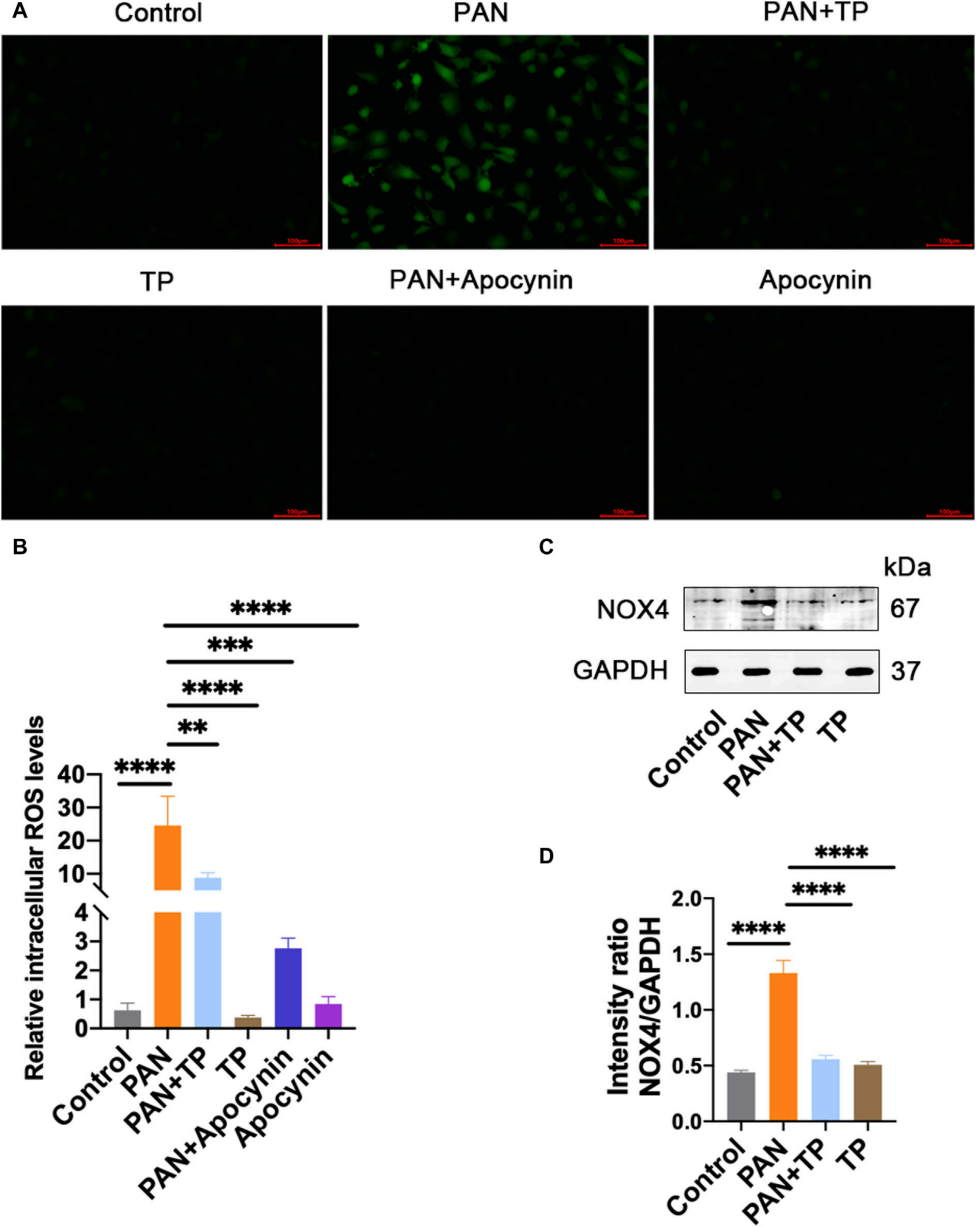
TP reduces PAN-induced increase in cellular ROS and NOX4. **(A)** The cellular ROS level was detected using DCFH-DA staining. Scale bar, 100 μm. **(B)** Quantification of ROS level. **p < 0.01, ***p < 0.001, ****p < 0.0001. **(C)** The protein expression of NOX4 in each group. **(D)** Relative protein level was calculated by the band intensity against GAPDH, respectively. ****p < 0.0001.

Many studies have shown that PAN-induced podocyte injury is associated with the overproduction of ROS [8]. Considering that podocytes express various NADPH oxidase subunits, including NOX4, we determined the cellular NOX4 and ROS levels. The results showed that PAN significantly increased the cellular ROS level and NOX4 expression (Figures 2A, C). However, TP significantly reduced the ROS level and NOX4 expression, which was confirmed by quantitative analysis (Figures 2B, D). Apocynin, an inhibitor of NADPH oxidase activity, led to a significant reduction in the ROS level induced by PAN. These results correlate with the changes in podocyte permeability, and indicate that PAN-induced oxidative stress might be involved in the increased podocyte permeability.

### TP upregulates expression of demethylase TET2

**FIGURE 3:**
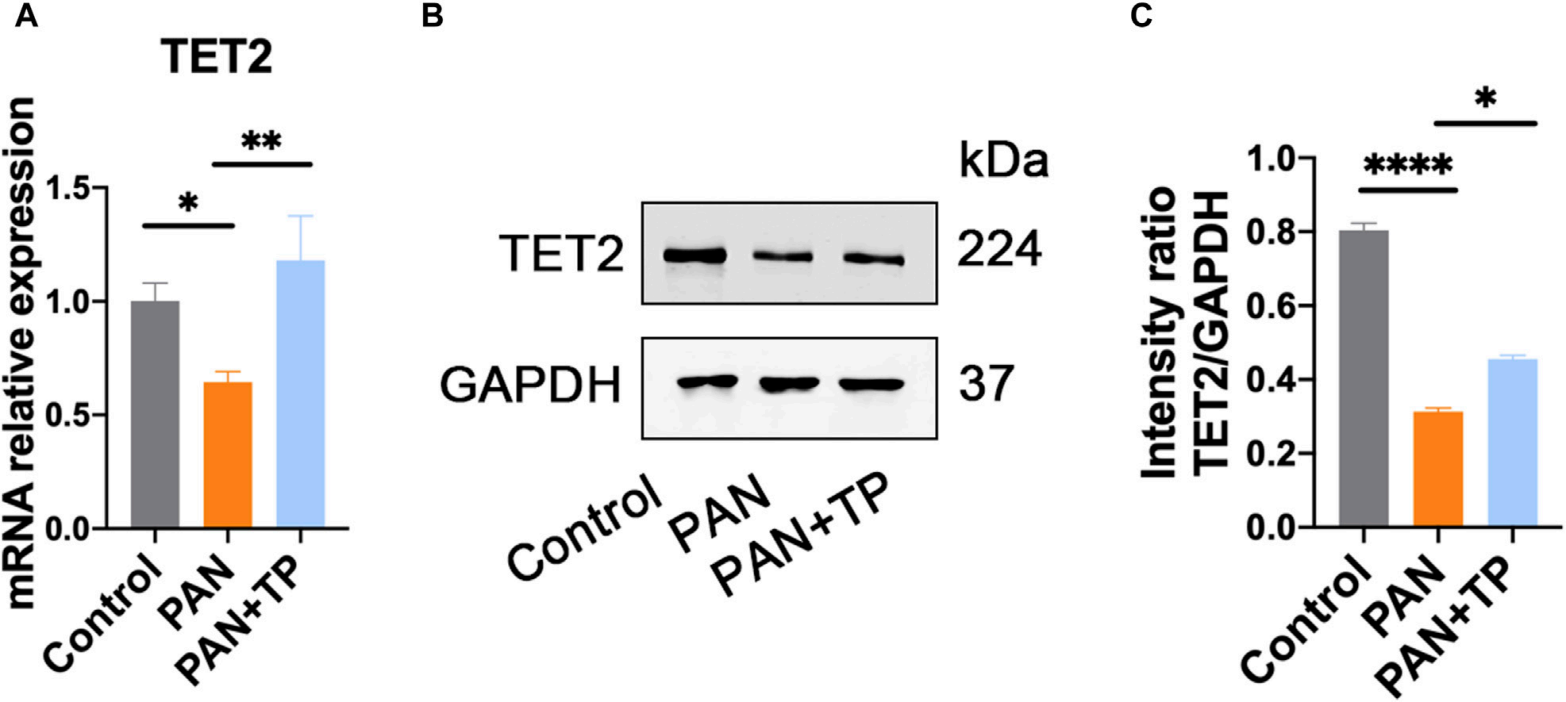
TP upregulates TET2 expression in PAN-treated podocytes. **(A)** The mRNA expression of TET2 in each group. Data are shown as mean ± SD. *p < 0.05, **p < 0.01. **(B)** The protein expression of TET2 in each group. **(C)** Relative protein level was estimated by the band intensity against GAPDH, respectively. *p < 0.05, ****p < 0.0001.

Growing evidence has shown that epigenetics played an important role in the evolvement of podocyte injury. Therefore, we detected the expression of DNA demethylase TET family proteins. qRT-PCR and western blotting results indicated that the expression of TET2 was significantly reduced in the PAN group compared with the control group (Figure 3). After TP treatment, the expression of TET2 increased significantly (Figure 3). However, TET2 and TET3 demonstrated no significant differences among the groups (data not shown). These results indicate that TP may selectively regulate the expression of TET2 in PAN-induced podocyte injury model.

### ZO-1 expression is regulated by epigenetics

**FIGURE 4:**
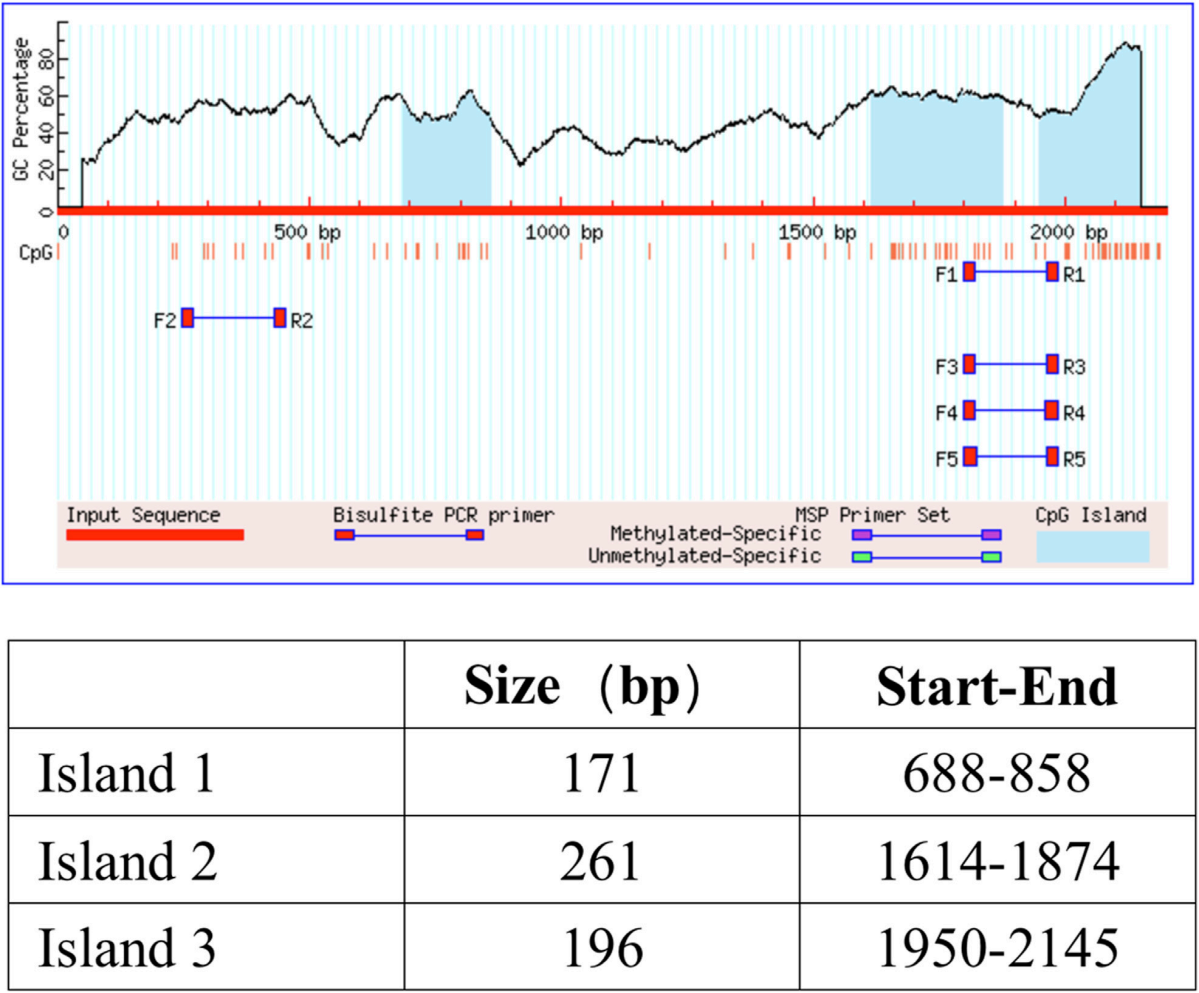
CpG island prediction results. Three CpG islands were found in the promoter region of ZO-1. The criteria used were as follows: Island size >100, GC Percent >50.0, and Obs/Exp >0.6.

**FIGURE 5:**
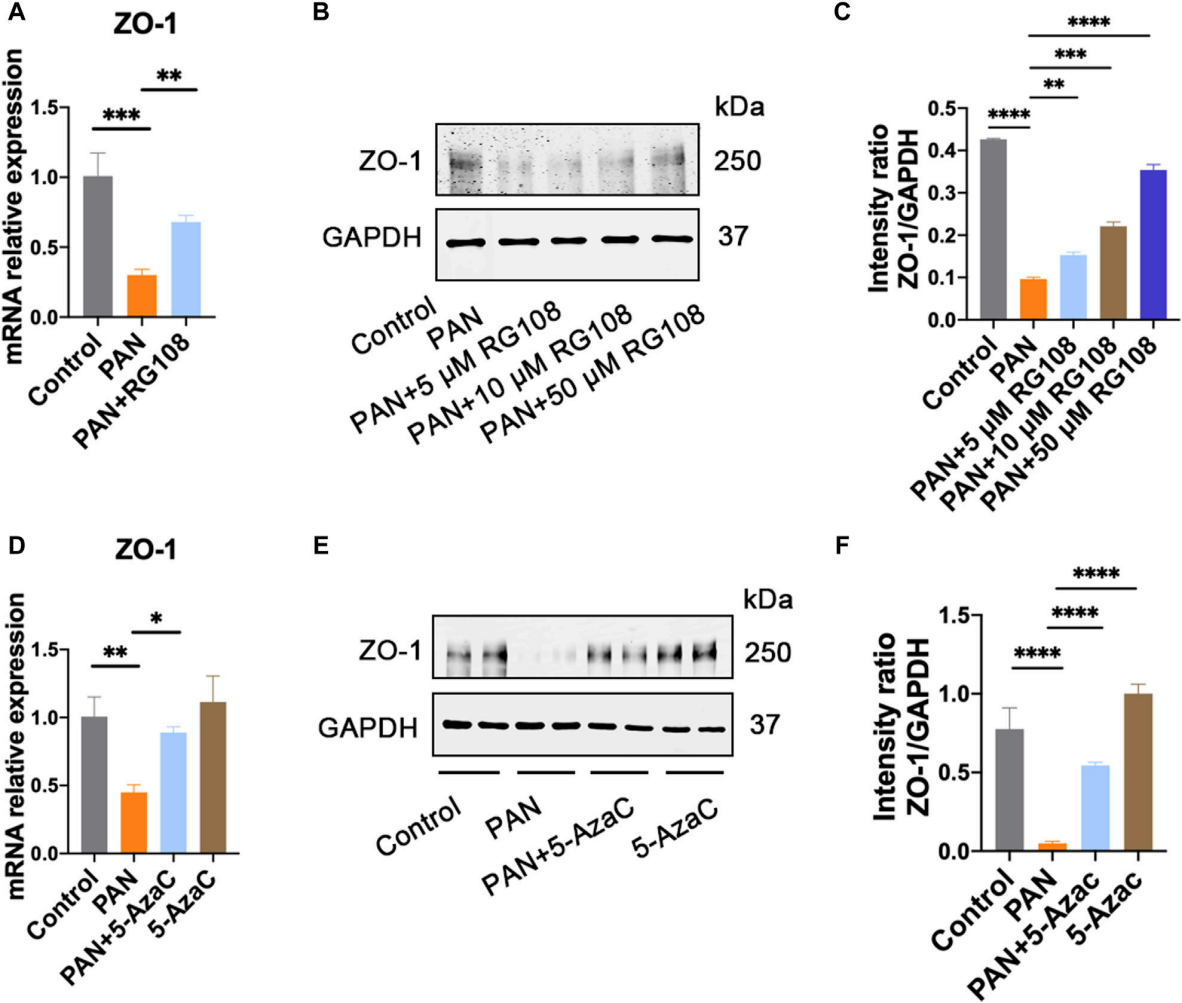
ZO-1 expression is regulated by epigenetics. **(A)** The expression of ZO-1 in each group was determined by qRT-PCR. Data are shown as mean ± SD. **p < 0.01, ***p < 0.001. Control: control podocyte; PAN: podocyte treated with 50 μg/mL PAN; PAN + RG108: podocyte treated with 50 μg/mL PAN and RG108 (5 μM, 10 μM, and 50 μM). **(B)** The expression of ZO-1 in each group was determined by western blot. **(C)** Relative protein level was calculated by the band intensity against GAPDH, respectively. **p < 0.01, ***p < 0.001, ****p < 0.0001. **(D)** The expression of ZO-1 in each group was determined by qRT-PCR. Data are shown as mean ± SD. Control: control podocyte; PAN: podocyte treated with 50 μg/mL PAN; PAN+5-AzaC: podocyte treated with 50 μg/mL PAN and 10 μM 5-AzaC; 5-AzaC: podocyte treated with 10 μM 5-AzaC. *p < 0.05, **p < 0.01. **(E)** The expression of ZO-1 in each group was determined by western blot. **(F)** Relative protein level was calculated by the band intensity against GAPDH, respectively. ****p < 0.0001.

It is well known that the TET family proteins play a crucial role in passive and active demethylation [17]. The synchronous upregulation of TET2 and ZO-1 suggests that ZO-1 expression may be regulated by TET2. To test our hypothesis, we firstly obtained the promoter region of ZO-1 by retrieving the upstream 2,200 bp sequence of the transcription start site from the human genome. Using the online methylation analysis software MethPrimer^1^, we found that the ZO-1 promoter region contains three CpG islands (688–858 bp, 1,614–1,874 bp, and 1,950–2,145 bp; Criteria used: Island size >100, GC Percent >50.0, Obs/Exp >0.6), indicating that the expression of ZO-1 may be epigenetically regulated (Figure 4). Subsequently, we used the DNA methyltransferase inhibitors 5-azacytidine (5-AzaC) and RG108 to treat the podocytes. The results showed that ZO-1 expression was significantly increased in podocytes treated with 5-AzaC and RG108 compared with the PAN alone stimulated group (Figure 5). Taken together, these results suggest ZO-1 expression is closely related to epigenetic regulation.

### TP can regulate the methylation status of the ZO-1 gene promoter

**FIGURE 6:**
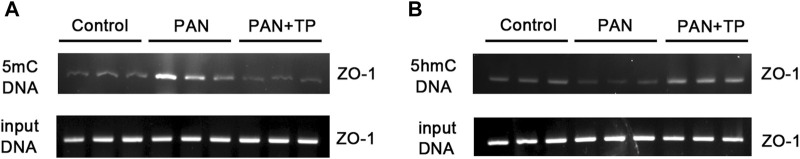
TP regulates the methylation status of the ZO-1 gene promoter. **(A)** Agarose gel electrophoresis images of the ZO-1 promoter amplified by polymerase chain reaction of methylated DNA obtained by methylated DNA immunoprecipitation (MeDIP). **(B)** Agarose gel electrophoresis images of the ZO-1 promoter amplified by polymerase chain reaction of hydroxymethylated DNA obtained by hydroxymethylated DNA immunoprecipitation (hMeDIP). The input DNA was used as the control for equal loading.

To further test whether TP regulates the methylation status of the ZO-1 promoter, we used MeDIP and hMeDIP methods to analyze the methylation status of ZO-1 in genomic DNA samples isolated from podocytes. As expected, the decreased expression of ZO-1 in the PAN group was associated with increased promoter methylation, while the promoter methylation levels of ZO-1 in the TP treatment group were significantly reduced (Figure 6A). Furthermore, we observed that ZO-1 demethylation upon TP intervention corresponded with increased ZO-1 hydroxymethylation (Figure 6B), suggesting that TP can mediate ZO-1 demethylation, which is accompanied by ZO-1 hydroxymethylation.

### TP upregulates the expression of ZO-1 through TET2

**FIGURE 7:**
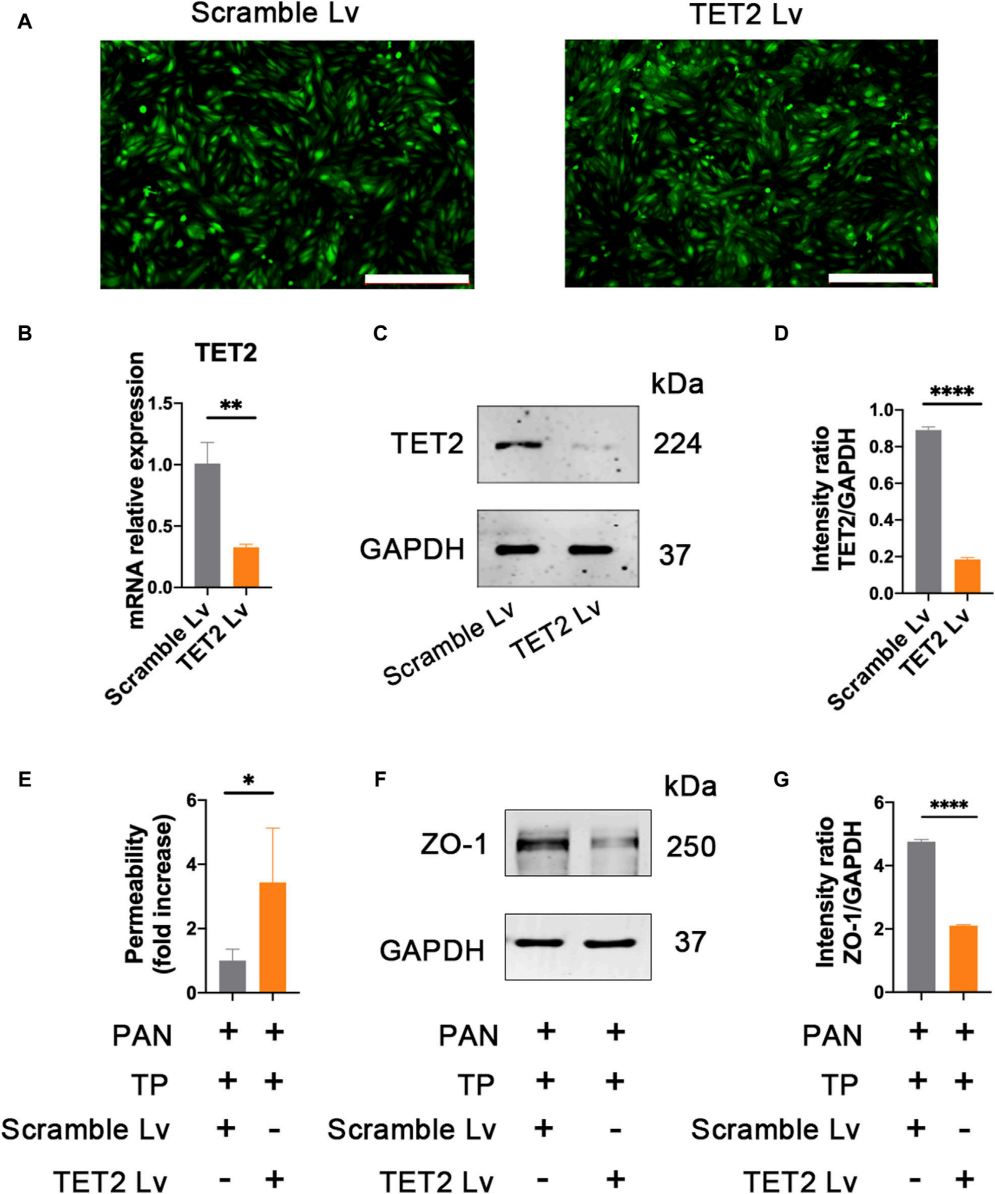
Knockdown *TET2* affects the expression of ZO-1. **(A)** The infection rates of lentivirus (Lv) carrying TET2 shRNA and scarmble Lv were evaluated by observing GFP fluorescence under a fluorescence microscope. Scale bar, 200 μm. **(B)** Efficacy of the Lv targeting TET2 was evaluated by qRT-PCR. Podocytes treated with TET2 Lv and scramble Lv were collected at 72 h **p < 0.01. **(C)** Efficacy of the Lv targeting TET2 was assessed by western blot. **(D)** Relative protein level was calculated by the band intensity against GAPDH, respectively. ****p < 0.0001. **(E)** Podocyte permeability was measured using FITC-labeled bovine serum albumin in a transwell chamber. *p < 0.05. **(F)** Expression of ZO-1 was detected after knocking down of *TET2*. Lv targeting TET2 and scramble Lv were transfected into the podocytes, respectively. After 72 h of transfection, podocytes were treated with PAN (50 μg/mL) and TP (5 nM). Cells were collected for western blotting analyses after 48 h of incubation. **(G)** Relative protein level was calculated by the band intensity against GAPDH, respectively. ****p < 0.0001.

**FIGURE 8:**
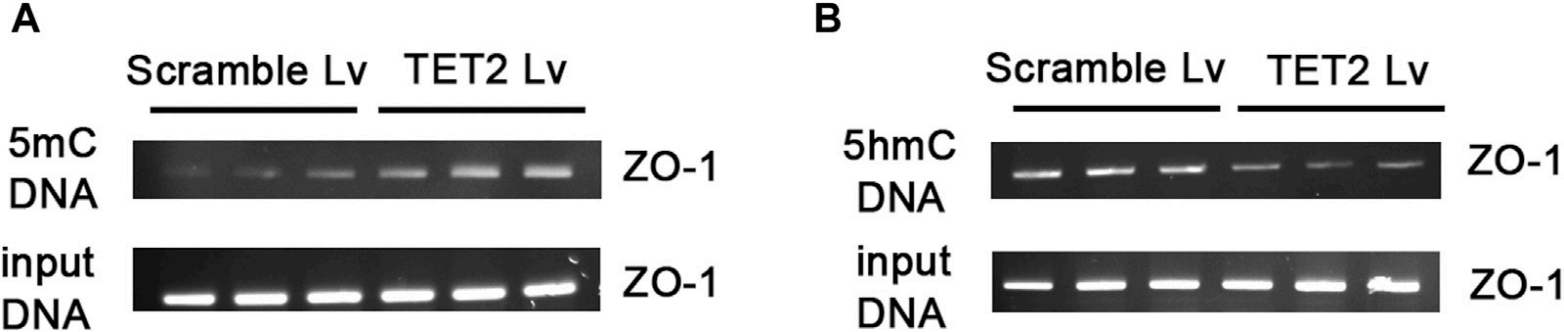
Knockdown *TET2* affected the methylation level of ZO-1. **(A)** Agarose gel electrophoresis images of ZO-1 promoter amplified by polymerase chain reaction of methylated DNA obtained by MeDIP. **(B)** Agarose gel electrophoresis images of ZO-1 promoter amplified by polymerase chain reaction of hydroxymethylated DNA obtained by hMeDIP. The input DNA was used as the control for equal loading.

In order to determine the role of TET2 in ZO-1 expression and methylation, we knocked down *TET2* using a lentivirus-mediated shRNA prior to TP intervention. After 72 h of infection, most podocytes strongly expressed GFP, indicating strong infectivity of the Lv (Figure 7A). qRT-PCR results showed the mRNA expression of TET2 in the TET2 Lv treatment group decreased by approximately 67.57% compared to that in the control group (Figure 7B). Western blot results also revealed that TET2 was significantly inhibited after Lv treatment (Figures 7C, D). The podocyte permeability increased significantly in the *TET2* knockdown group compared with the scrambled Lv group (Figure 7E). Moreover, the expression of ZO-1 was decreased significantly in the *TET2* knockdown group compared with the scrambled Lv group, even after treatment with TP (Figures 7F, G). Meanwhile, compared with the control group, increased promoter methylation of ZO-1 was observed in the *TET2* knockdown group (Figure 8A), accompanied by decreased ZO-1 hydroxymethylation (Figure 8B). Altogether, these results indicate TP may upregulate the expression of ZO-1 through TET2-mediated 5 mC demethylation.

## Discussion

Growing evidence shows that TP has a strong antiproteinuric effect through multiple mechanisms. Notably, the current studies on TP intervention in podocyte injury at home and abroad mainly focus on the changes in the podocyte phenotypic molecules and the related signaling pathways. Few studies have considered the perspective of epigenetics. This study explored the relationship between TP, TET2, and ZO-1 from the novel perspective of epigenetics.

ZO-1 is a tight junction protein which plays an essential role in maintaining the integrity of the podocyte filtration barrier [18–20]. Previous studies have demonstrated that podocyte-specific depletion of ZO-1 leads to damage to SD integrity, thus causing proteinuria [18]. Besides, ZO-1 can not only regulate the stability of the SD protein nephrin but can also regulate cytoskeleton organization by binding to F-actin [20–22]. Therefore, clarification of the regulation mechanism of ZO-1 holds considerable clinical importance.

TET proteins mediate DNA demethylation by converting 5-methylcytosine (5 mC) to 5-hydroxymethylcytosine (5 hmC). We have previously shown that TET1–3 have a differential expression pattern in the podocytes, with relatively high expression of TET2 and low expression of TET1 and TET3 [23]. Other studies have also confirmed the expression of TET2 and TET3 is higher than that of TET1 in the kidney [24]. Furthermore, we found TET2 was downregulated in the TGF-β1-induced podocyte injury model, and TP could reverse podocyte EMT by TET2-mediated podocyte SD gene demethylation. TET2 was consistently demonstrated to play an important role in both cisplatin-induced and ischemia reperfusion-induced acute kidney injury [24–26]. These findings studies suggest that TET2 plays an important role in the pathophysiological processes in the kidney.

In this study, multi-level experiments showed a correlation between TET2 and ZO-1 expression. Firstly, TET2 expression synchronized with ZO-1 expression in the PAN-induced podocyte injury model and TP intervention group. Secondly, three predicted CpG islands were found in the promoter of ZO-1 using MethPrimer online analysis software. Treatment with 5-AzaC and RG108 upregulated ZO-1 expression compared with the PAN-induced group. Thirdly, increased ZO-1 promoter methylation level, decreased ZO-1 expression level, and increased podocyte permeability were found in the *TET2* knockdown group compared with the scrambled Lv group, even after treatment with TP. All these findings indicate that TET2-mediated ZO-1 promoter demethylation reverses podocyte injury.

Currently, there are conflicting reports on the toxicity and therapeutic effects of TP on the kidney [10, 27, 28]. TP showed renoprotection effects against metronidazole-induced injury in zebrafish, puromycin-induced nephropathy, PM 2.5-induced podocyte injury, and deoxycorticosterone acetate-salt hypertension-induced renal injury in mice subjected to uninephrectomy [13, 29, 30]. In contrast, TP was also shown to exert nephrotoxicity in normal BALB/c mice and renal tubular epithelial cell lines NRK-52E and HK-2 [31–33]. Besides, Lixin Sun *et al.* found that TP-induced nephrotoxicity in rats leads to changes in the barrier and permeability of the proximal tubular epithelium by altered localization of ZO-1 [32]. Similarly, Huang Shan *et al.* demonstrated that TP caused decreased expression and abnormal subcellular localization of ZO-1 in a TP-induced cholestasis model [34]. In contrast, our study provides evidence that TP has a protective effect on PAN-induced podocyte injury through the upregulation of ZO-1 expression and decreased podocyte permeability. These contradictory effects may be attributed to various factors, including the dosage of TP and differences among the experimental subjects. Research on TP nephrotoxicity mostly focuses on normal cells, while its protective effect is mostly investigated in damaged cells. Taken together, these results indicate that TP is a double-edged sword, and only its correct and standardized use can avoid the side effects.

The limitation of our study is that we mainly focused on the cellular level to explore the epigenetic regulation of podocyte tight junction protein ZO-1 by of TP. We plan to use a CKD-related animal model to reveal the exact molecular mechanism and provide more *in vivo* evidence in the future.

In summary, the present study shows that TP can reverse ZO-1 expression by regulating TET2-mediated DNA demethylation. TET2 could be a promising target for developing a treatment strategy for podocyte injury. Our observations may also provide new ideas for further studies on the mechanism underlying the therapeutic effect of TP on CKD.

## Data Availability

The original contributions presented in the study are included in the article/supplementary material, further inquiries can be directed to the corresponding author.

## Footnotes

1
http://www.urogene.org/methprimer/
